# Biomimetically Engineered Demi‐Bacteria Potentiate Vaccination against Cancer

**DOI:** 10.1002/advs.201700083

**Published:** 2017-06-15

**Authors:** Dezhi Ni, Shuang Qing, Hui Ding, Hua Yue, Di Yu, Shuang Wang, Nana Luo, Zhiguo Su, Wei Wei, Guanghui Ma

**Affiliations:** ^1^ State Key Laboratory of Biochemical Engineering Institute of Process Engineering Chinese Academy of Sciences 1 North 2nd Street Zhongguancun, Haidian District Beijing 100190 P. R. China; ^2^ University of Chinese Academy of Sciences No. 19A Yuquan Road Beijing 100049 P. R. China; ^3^ Molecular Immunomodulation Laboratory School of Biomedical Sciences Monash University Clayton Victoria 3800 Australia; ^4^ Jiangsu National Synergetic Innovation Center for Advanced Materials Nanjing 211816 P. R. China

**Keywords:** bacteria, biomimetic engineering, cancer immunotherapy, particulate adjuvant, vaccines

## Abstract

Failure in enhancing antigen immunogenicity has limited the development of cancer vaccine. Inspired by effective immune responses toward microorganisms, demi‐bacteria (DB) from *Bacillus* are engineered as carriers for cancer vaccines. The explored hydrothermal treatment enables the *Bacillus* to preserve optimal pathogen morphology with intrinsic mannose receptor agonist. Meanwhile, the treated *Bacillus* can be further endowed with ideal hollow/porous structure for efficient accommodation of antigen and adjuvant, such as CpG. Therefore, this optimal engineered nanoarchitecture allows multiple immunostimulatory elements integrate in a pattern closely resembling that of bacterial pathogens. Such pathogen mimicry greatly enhances antigen uptake and cross‐presentation, resulting in stronger immune activation suitable for cancer vaccines. Indeed, DB‐based biomimetic vaccination in mice induces synergistic cellular and humoral immune responses, achieving potent therapeutic and preventive effects against cancer. Application of microorganism‐sourced materials thus presents new opportunities for potent cancer therapy.

## 1 Introduction

Vaccination possesses particular advantages and promising potential for future cancer treatment. However, the poor immunogenicity of tumor antigens has obstructed the development of cancer vaccine. In a phase II clinical trial, the gp100 peptide vaccine used alone had little antitumor activity.^1^ Rosenberg et al. also reported that the overall clinical response rate of cancer peptide vaccines was extremely low (only 2.6% among 440 cancer patients).^2^ To solve this problem, great efforts have been devoted to exploit novel immunomodulators or vaccine adjuvants.

It is well known that the immune system can be efficiently evoked by pathogens. The strong immunogenicity of pathogenic microorganisms can be attributed to pathogen‐associated molecular patterns (PAMPs), such as intracellular cytidine‐phosphate‐guanosine (CpG) oligodeoxynucleotides and surface polysaccharide derivatives.^3^, ^4^ As essential functional components, these PAMPs are shared among microbes and possess conserved molecular structures. By recognizing these structures, the host immune system can rapidly distinguish “nonself” from “self” and activate adaptive immunity.^5^ This knowledge has inspired the use of PAMPs in vaccine development to enhance immune responses.^6^ However, owing to the extreme complexity of the immune system and the in vivo environment (e.g., metabolization, cell population, and cytokines), unpredictable and elusive outcomes often result, especially, when more than one PAMP element is used in combination.^7^, ^8^ In addition to natural PAMP signals, recent studies indicate that unique physical properties (e.g., size, shape, and texture) also make pathogens conspicuous to the immune system. For example, Doshi and Mitragotri revealed that the size of 2–3 µm (most commonly found in natural bacteria) exhibited the highest attachment to macrophage.^9^ Another study suggested that rod‐like particles enjoyed an appreciable advantage for their internalization.^10^ The highly repetitive arrays of antigen epitopes on the particles were also demonstrated to trigger strong humoral immune response, as the B cells have evolved to recognize dense, repetitive epitope arrangements on the surfaces of pathogens (e.g., viruses and flagellum).^11^ All these findings suggest that a biomimetic vaccine with pathogenlike PAMP presentation and physical properties may be a better choice for successful vaccine development. Unfortunately, realization of this vaccine remains a formidable task, as complicated engineering and elaborate fabrication will be involved.^12^, ^13^, ^14^

**Figure 1:**
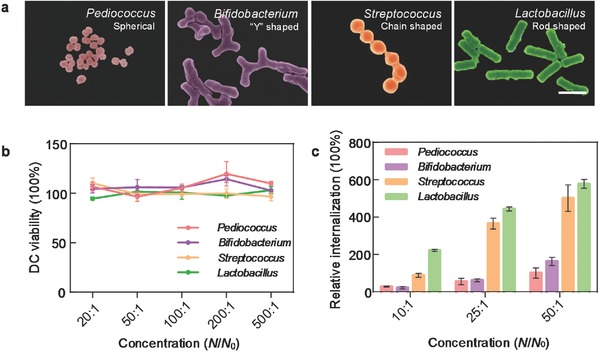
Effect of bacterial shape on DC internalization. a) Bacteria with different shapes after hydrothermal treatment. Scale bar is 1 µm. b) In vitro biocompatibility evaluation of different bacteria after hydrothermal treatment. c) Dose‐dependent internalization of these treated bacteria with DCs after a 24 h incubation period at 37 °C. *N*: the total number of particles we dosed, *N*_0_: the total number of DCs. The data in parts (b) and (c) represent the mean ± SD of three independent experiments with *n* = 3.

**Figure 2:**
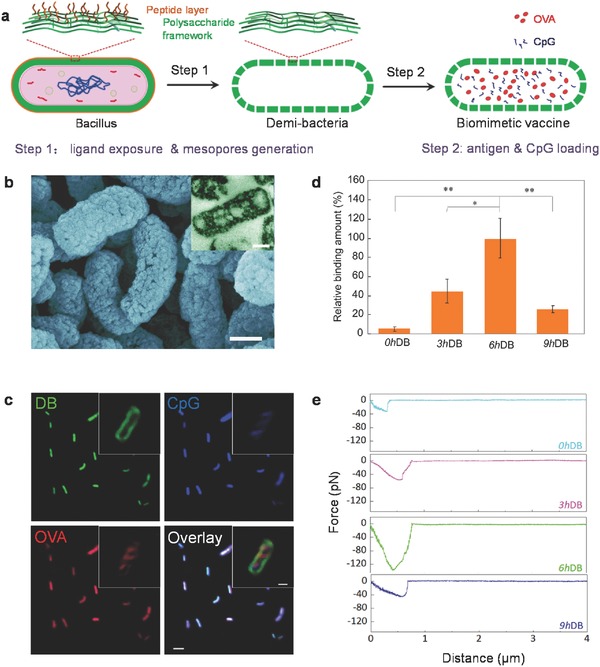
Fabrication of DB and biomimetic vaccine. a) Schematic illustration of the synthesis of DB and biomimetic vaccine construction. b) Scanning electron microscope (SEM) with inserted transmission electron microscope (TEM) images of DB. Both scale bars were 200 nm. c) High‐resolution CLSM images showing that FITC‐OVA (red) and AMCA‐CpG (blue) were encapsulated inside HiLyte Fluor 647‐conjugated DB (green). Scale bar is 2 µm. Scale bar in the inset is 200 nm. d) Comparison of the CD206 binding of DB prepared with different hydrothermal treatment time. Data in the figure represent the mean ± SD with *n* = 3. *p < 0.05, **p < 0.01. e) Representative profiles of the affinity force between DCs and DB prepared with different hydrothermal treatment times.

Herein, by selectively reserving and exposing bacterial immunostimulatory features both in morphology and PAMP signal aspects, we developed a novel method to fabricate bacterial pathogen‐mimic vaccine backbones (demi‐bacteria, DB). In this case, biomimetic vaccine of excellent safety and ability to elicit strong antigen‐specific immune response can thus be facilely constructed. Briefly, DB with optimal shape (**Figure** **1**) were transformed from *Lactobacillus casei* via fine‐tuned hydrothermal treatment (**Figure** **2**a, step 1). These DB thus inherited both surface ligands and pathogen‐like physical properties from their archetypes. Subsequently, antigen and CpG could be efficiently loaded into the DB, which had a hollow and porous structure (Figure 2a, step 2). In this way, different PAMPs were reasonably arranged in a pattern adopted by bacterial pathogens. These biomimetic features were intended to enhance subsequent antigen delivery/presentation and immune activation. Using different tumor‐bearing models, we systematically verified the feasibility of the as‐designed biomimetic vaccine for cancer therapy and prophylaxis.

## 2 Results and Discussion

### 2.1 Preparation of Optimal DB and Construction of Biomimetic Vaccine

Several reports have addressed the importance of bacterial shape, which can play an important role in cellular recognition and internalization during the infection process.^9^, ^15^ In this aspect, deeper insights into the shape‐dependent internalization by antigen present cells (APCs) can help us to design a better vaccine platform. To this end, we selected four kinds of lactic acid bacteria (*Pediococcus acidilactici*, *Streptococcus thermophilus*, *Lactobacillus casei*, and *Bifidobacterium adolescentis*) with distinguishing shapes (spherical, chain shaped, rod shaped, and “Y” shaped) and treated them with the hydrothermal process for the investigation of dendritic cell (DC) uptake profiles. After optimized hydrothermal treatment (Figure S1, Supporting Information), fragile cellular components (e.g., cell membrane, intracellular protein, and genetic materials) and pretreatment chemical reagents such as formaldehyde and Triton X‐100 were completely hydrolyzed and removed,^16^ whereas the cell wall framework, with its solid structure, could be well preserved.^17^ So, the obtained biocompatible bacterial candidates (Figure 1b) not only kept their original shapes (Figure 1a) but also had similar surface properties (Table S1, Supporting Information), which paved the way for subsequent comparative investigation. As shown in Figure 1c, the internalization amount increased in the sequence of *Pediococcus*, *Bifidobacterium*, *Streptococcus*, and *Lactobacillus* at a series of concentrations. A possible explanation for such a shape‐dependent internalization behavior could be attributed to the different high aspect ratio of these bacteria. As previously reported,^18^ exogenous particles might be internalized only when the target was encountered at sites that presented a local shape with an angle Ω below a critical value of 45°. Ω was defined as the angle between the membrane normal and the line defining the local particle curvature at the point of cell contact. The smaller the value of Ω was, the faster the particles were internalized. According to our measurement (Figure S2, Supporting Information), the value of Ω for these treated bacteria decreased in the sequence of *Pediococcus*, *Bifidobacterium*, *Streptococcus*, and *Lactobacillus*. In this case, *Lactobacillus* with the smallest value of Ω induced the best DC internalization.

Having discovered the preferred shape, we next generated mesopores (Figure 2a,b) in the *Lactobacillus* shell for subsequent loading by further optimization of our hydrothermal treatment (Figure S3a, Supporting Information). High‐resolution confocal laser scanning microscope (CLSM) images showed that the CpG and Ovalbumin (OVA) molecules resided inside the DB (Figure 2c), demonstrating successful encapsulation. The ideal hollow/porous architecture led to efficient loading of OVA and CpG at 85.2 and 7.5 wt%, respectively (Figure S3b, Supporting Information). The resulted DB platform also showed good stability (Figure S3c, Supporting Information) and desired sustained release profiles (Figure S3d, Supporting Information).

We also noted that the cell wall substance of *Lactobacillus* is mainly composed of *N*‐acetylglucosamine, an identified ligand that can promote efficient recognition and endocytosis by APCs via an mannose receptor (MR)‐mediated pathway.^1^ Thus, the possibility of inheriting these PAMPs together with the cell wall framework exists. However, this pathogen‐like feature is disguised, as a peptide layer covers the outmost surface of *Lactobacillus*.^19^ Fortunately, the exterior peptide can be removed by sustaining hydrolysis during the hydrothermal process. As shown in Figure 2d, the capacity to bind the recombinant MR (CD206) of the DB increased with the hydrothermal treatment time until 6 h (6hDB), as more ligands were exposed. By contrast, further treatment (9 h) decreased MR binding, probably owing to ligand dysfunction caused by excessive carbonization.^20^ Similarly, affinity between the 6hDB and DCs was found to be the strongest (Figure 2e), indicating that DCs exhibited favorable recognition of and binding to the DB with sufficient MR ligands. Considering their superior MR ligand exposure, the 6hDB were selected for subsequent research.

### 2.2 Biomimetic Features Induce Infection‐Like DC Internalization via early Endosomal Pathway

**Figure 3:**
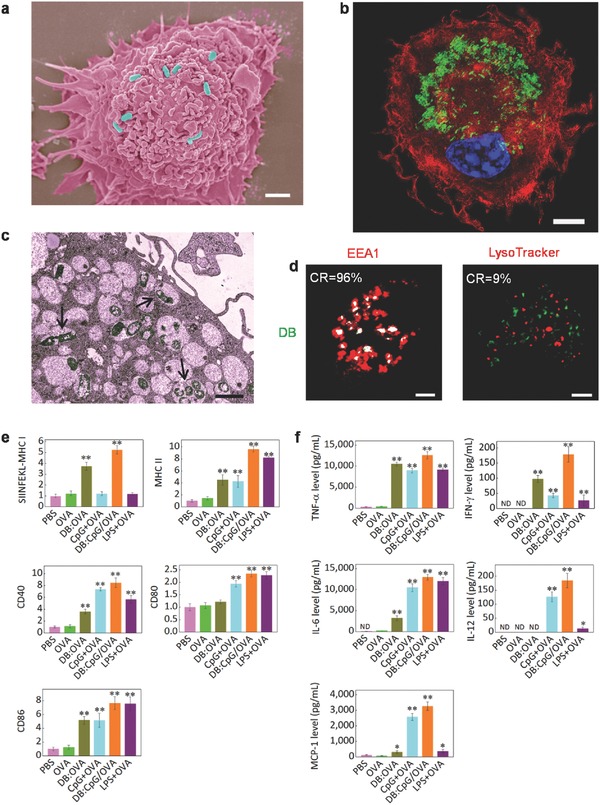
DC internalization of DB and in vitro DC stimulation by biomimetic vaccine. a) The SEM image of a DC after coincubation with DB. Scale bar is 2 µm. b) The CLSM image of a DC “invaded” by high amounts of DB (green) after incubation with 2.5 µg mL^−1^ DB for 24 h. Scale bar is 5 µm. c) The TEM image showing that DB settled inside certain organelles (as the arrows indicated) after internalization by DCs. Scale bar is 1 µm. d) The internalized DB were confined to EEA1^+^ early endosomes (left panel), rather than to lysosomes (right panel). The CR values denote the colocalization ratio of DB to the given organelles. Scale bar is 5 µm. e) Expression of recognition signals (SIINFEKL‐MHC I and MHC II) and costimulatory markers (CD40, CD80, and CD86) on DCs after 24 h of incubation with 1 µg mL^−1^ soluble OVA or an equivalent dose of OVA loaded with or without 100 ng mL^−1^ CpG into DB (2.5 µg mL^−1^). A mixture of lipopolysaccharide (100 ng mL^−1^) plus OVA (LPS + OVA) was used as a positive control. The data were normalized to the expression level of the Phosphate‐buffered saline (PBS) group. f) Cumulative cytokine secretion of DCs during the 24 h of incubation with different formulations. Data in parts (e) and (f) represent the mean ± SD of three experiments with *n* = 3. *p < 0.05, **p < 0.01.

Owing to their remarkable mutual affinity, the DB were identified as pathogens by APCs and induced rapid endocytosis. By trapping the internalization moment of DB, we found that DCs forcefully seized DB individuals (**Figure** **3**a). In this case, the DB could enter APCs at an extremely high rate (Figure 3b; Figure S4a, Supporting Information), which is distinctly superior to traditional particular adjuvants (Figure S4b, Supporting Information). Such an efficient DC uptake of DB, as expected, greatly enhanced the intracellular delivery of both OVA and CpG (Figure S4c, Supporting Information). Once the MRs were blocked, the internalization decreased by 51.1% (Figure S4d, Supporting Information), again confirming the significant role of MR ligands during antigen uptake. The surficial pathogen‐like feature also influenced the intracellular trafficking of the DB. As shown in Figure 3c,d, the DB mainly settled inside early endosomes, rather than inside lysosomes, which is consistent with previous reports on MR‐mediated uptake.^21^ It is also worth mentioning that these DB exhibited no influence on the viability of APCs (data not shown), although drastic internalization did occur.

### 2.3 The Biomimetic Vaccine Potently Activates DCs

We next evaluated the effect of the as‐constructed biomimetic vaccine on DC activation and antigen presentation. Compared with DCs pulsed with soluble OVA, DCs treated with OVA‐loaded DB (DB:OVA) presented epitope peptides (SIINFEKL, OVA257‐264) with greatly enhanced efficiency via the major histocompatibility complex class I (MHC I)‐restricted pathway (Figure 3e; Figure S5a, Supporting Information). This result is partly attributable to the MR‐mediated intracellular trafficking pathway, which induces effective cross‐presentation.^18^ Once CpG was codelivered (DB:CpG/OVA), the most robust upregulation (up to 525.1%) of the SIINFEKL–MHC I complex was detected (Figure 3e), owing to the immunomodulatory effect of CpG via triggering toll‐like receptor 9 (Figure S6, Supporting Information).^22^ Similarly, the levels of surface MHC II molecules and the costimulatory markers CD40, CD80, and CD86 were significantly increased by the pulse of DB:CpG/OVA (Figure 3e; Figure S5a, Supporting Information), indicating the effective activation of DCs. Moreover, invasion by DB:CpG/OVA induced DCs to secrete immunomodulatory cytokines and chemokines, including interleukin‐12 (IL‐12), interferon‐γ (IFN‐γ), tumor necrosis factor (TNF‐α), IL‐6, and monocyte chemotactic protein‐1 (MCP‐1), in very large amounts (Figure 3f; Figure S5b, Supporting Information), which were proposed to greatly benefit the activation and differentiation of CD8 and CD4 T cells.

### 2.4 Antigen Deposition and Programming of APC Trafficking In Vivo

**Figure 4:**
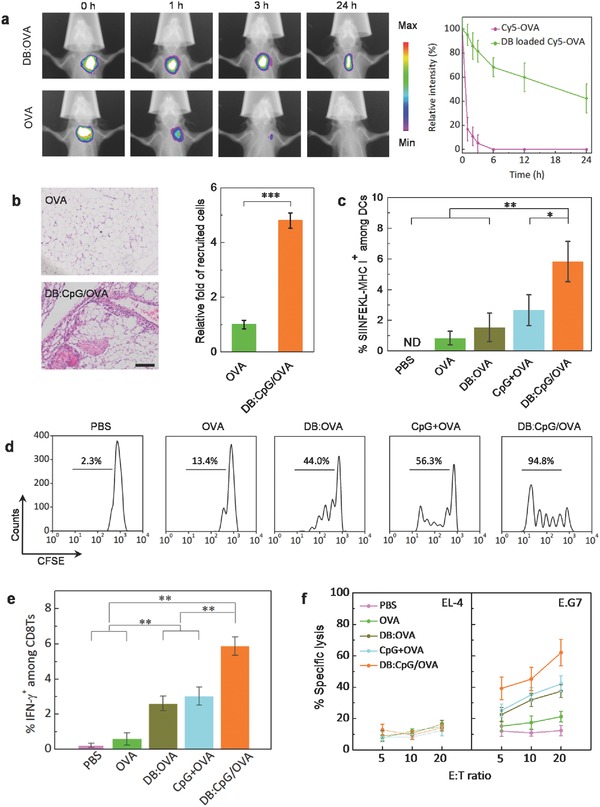
Biomimetic vaccine enhances in vivo antigen deposition and elicits potent CD8T responses. a) Comparison of the distribution of DB‐loaded Cy5‐OVA and free Cy5‐OVA after subcutaneous administration in the hind neck of C57BL/6 mice; the white zone in the image indicates that the fluorescence intensity exceeded the maximum. b) Histological images and the corresponding analysis of monocyte infiltration at the injection site after 24 h. Scale bar is 200 µm. c) Frequency of SIINFEKL–MHC I+ DCs in the draining lymph nodes after injecting different vaccine formulations. d) FC analysis showing that after being adoptively transferred into the recipient mice, which received varying formulations, CFSE‐labeled MHC I‐restricted, OVA‐specific T cells underwent proliferation to different extents. In each group, 20 µg of soluble OVA or an equivalent dose of OVA loaded with or without 2 µg of CpG in 50 µg of DB was subcutaneously injected. The gates on each histogram indicate the percentage of divided cells in each sample. e) Percentages of IFN‐γ‐secreting cells among splenic CD3^+^CD8^+^ T cells from mice 28 d postvaccination with different formulations. f) In vitro killing assay showing the percentage of specific lysis at various effector/target (E:T) ratios using EL‐4 cells (mock control) or E.G7 cells (specific target) after 12 h of incubation with the splenocytes of mice immunized with different formulations. Data in parts (b), (c), (e), and (f) represent the mean ± SD of three independent experiments with *n* = 3. *p < 0.05, **p < 0.01.

Having identified the potent abilities of the biomimetic vaccine in DC activation and antigen presentation, we continued to investigate the in vivo fate of antigens after encapsulation in DB. As shown in **Figure** **4**a, the near‐infrared (NIR) fluorescence of free Cy5‐OVA became nearly undetectable 3 h after administration, whereas encapsulation in DB resulted in antigen persistence at the injection site for 24 h, demonstrating the protection of the antigen from rapid metabolism and diffusion. Given that the biomimetic vaccine stimulated DCs to secrete large quantities of MCP‐1 (an APC‐recruiting chemokine), patrolling APCs could be recruited to the vaccine site in time (Figure 4b) via communication with local APCs. Together with the prolonged persistence of the antigen postinjection, such a temporospatial confluence could enhance the probability of APCs encountering the antigen inside a local “pathogen arsenal,” ensuring the accomplishment of efficient antigen delivery. Furthermore, in vivo tracking showed that, after their activation by the biomimetic vaccine, the antigen‐primed DCs could migrate to the draining lymph nodes (LNs) in greatly enhanced efficiency over that driven by sole OVA (Figure 4c). The presence of plenteous activated DCs made efficient antigen presentation to naive CD8 T cells (CD8Ts) being expected.

### 2.5 The Biomimetic Vaccine Arouses Strong Antigen‐Specific Cytotoxic T Cell Response

The generation of strong and specific cytotoxic T lymphocyte (CTL) responses is indispensable for cancer cell elimination. Thus, we continued to evaluate the abilities of different formulations to promote the proliferation and activation of OVA‐specific CD8Ts in vivo. As shown in Figure 4d, OVA alone only elicited very weak T‐cell proliferation. Although immunization with DB:OVA or CpG + OVA showed improved efficiency in CD8T cross‐priming, the most intensive proliferation was achieved when CpG and DB were used in combination, with 94.8% of CD8Ts having undergone division at 72 h (Figure 4d). This result suggested that the presence of DB and CpG in a complex form provided synergic stimulation that induced effective CD8T expansion. To further estimate the cytotoxic effect of the endogenous T‐cell response, the splenocytes of immunized mice were harvested 28 d postvaccination, and the percentage of IFN‐γ‐secreting CD8Ts was detected. Again, mice immunized with DB:CpG/OVA were found to have the highest percentage of IFN‐γ‐secreting CD3^+^CD8^+^ T cells (Figure 4e). As IFN‐γ is an important cytokine that actualizes the cytotoxicity of CTLs, this result demonstrated that the combined pathogen‐like features intensified the CTL response. As a result, after 12 h of incubation with the splenic CD8Ts of mice that received the DB:CpG/OVA vaccine, 62.1% of the murine lymphoma E.G7 cells (an OVA‐expressing derivative of EL‐4 cells) were lysed, whereas no evident damage to EL‐4 cells was detected (Figure 4f), indicating the effective and specific clearance of antigen‐positive targets.^23^

### 2.6 Effective Inhibition of Tumor Growth and Metastasis

**Figure 5:**
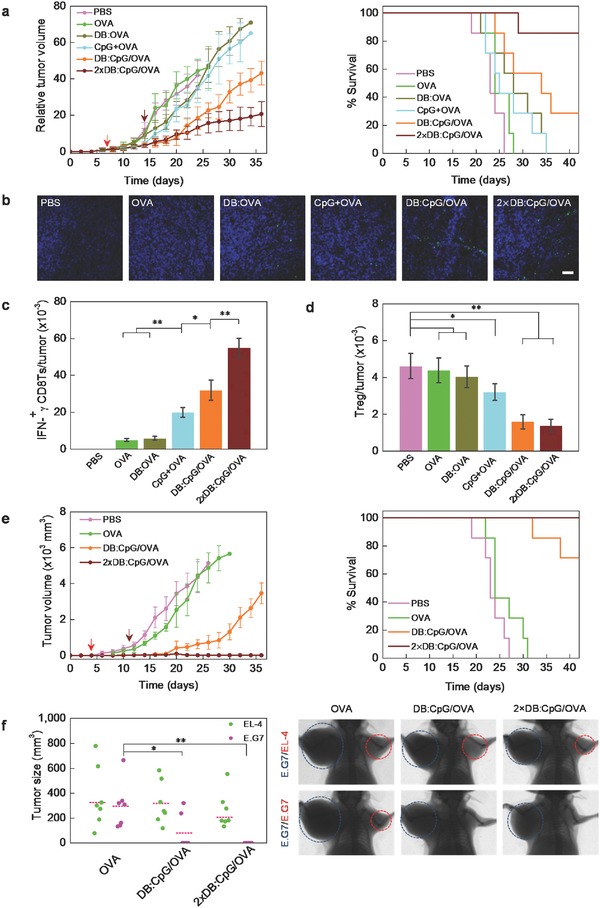
Biomimetic vaccine induces effective immune elimination against lymphoma. a) Tumor progress and the survival time of tumor‐bearing mice after treated with different formulations. Day 0 was the day of tumor transplantation, and arrows indicate days of prime (red) and booster (wine) vaccination, respectively. b) CLSM images of the infiltration of IFN‐γ secreting CTL (green) in tumor on day 21 after different treatments. Scale bar is 100 µm. c) Quantitative analysis of IFN‐γ‐secreting CD8^+^ T in tumor after mice treated with different formulations. d) Frequencies of regulatory T lymphocyte in tumor after mice treated with different formulations. e) Mice with early‐stage tumors were immunized with different formulations, and the tumor progress and survival times were monitored to evaluate the effect of early therapy. f) Antigen‐dependent protection against lymphoma metastasis. Tumor‐bearing mice received the same vaccination as in part (a) and were again challenged with 2 × 10^5^ E.G7 or EL‐4 cells in the right axillary region 7 d after the last treatment. The presented data are the tumor sizes at 10 d after the second challenge. The presented images are from individual mice with representative tumor sizes from each group. The primary and secondary tumors are emphasized by blue and red circles, respectively. Data in parts (a), (c), (d), and (e) represent the mean ± SD with *n* = 7. *p < 0.05, **p < 0.01.

After confirming the in vivo safety of the biomimetic vaccine (Table S2 and Figure S7 of the Supporting Information), we evaluated its anticancer efficiency in tumor models. To test the therapeutic effects on established tumors, we subcutaneously administered different vaccine formulations to mice with palpable E.G7 tumors (7 d after transplantation). As shown in **Figure** **5**a, vaccination with free OVA led to no noticeable tumor restriction. When DB or CpG was employed, only a slight delay in tumor growth was achieved, indicating the insufficiency of an individual element. After immunization with DB:CpG/OVA, significant delay on tumor growth was observed. However, uncontrolled tumor growth was observed from day 21, as rapid tumor development had outraced the immune response. To solve this problem, a booster immunization (2 × DB:CpG/OVA) was given on day 14. After the enhanced immunization, the aggressive development of malignant lymphoma could be controlled, and a greatly prolonged survival time was achieved. Immune response was proved to play a pivotal role during such a remarkable tumor inhibition, as efficient infiltration of CTL and population restriction of immune inhibitory Treg in tumor microenvironment were detected (Figure 5b–d). It is also noteworthy that tumor burden is usually associated with multiple organ dysfunction or failure, leading to a poor health condition in patients. To our surprise, determination of serum biochemical parameters showed that the serum aspartate aminotransferase, lactate dehydrogenase (LDH), alanine aminotransferase, and alkaline phosphatase concentrations of the DB:CpG/OVA group mice returned to normal ranges (Table S3, Supporting Information), reflecting a good health condition after immunotherapy with the biomimetic vaccine.^24^

Recent studies have opened the possibility of discovering cancer lesions at a very early stage.^25^, ^26^ To evaluate the therapeutic effect on tumors in an early stage, we next vaccinated mice on day 4 (transplantation on day 0). After a booster immunization of DB:CpG/OVA on day 11, the development of tumors was completely restricted, and no death occurred during the 6‐week observation period (Figure 5e). Regarding the greatly improved therapeutic effect achieved in the early‐stage tumor models, early immune intervention, before the establishment of a tumor‐associated immunosuppressive environment, is proposed to be significantly responsible.^27^ Thus, a malignant tumor can be curable if timely diagnosis and therapy with the biomimetic vaccine are adopted.

Metastasis is one of the most severe problems and leads to a poor prognosis. To test the antimetastatic effect of the biomimetic vaccine, we challenged tumor‐bearing mice with 2 × 10^5^ E.G7 cells at another axillary region 7 d after the last vaccination. In contrast to the complete failure in the OVA group, five of seven mice in the DB:CpG/OVA group were protected from tumor metastasis (Figure 5f). With a booster immunization, thorough prevention of E.G7 tumor outgrowth was achieved, indicating an excellent antimetastatic ability. As cancer recurrence caused by residual cancer cells and metastasis always results in the failure of chemotherapy or surgical excision, immunotherapy with the biomimetic vaccine would be a very potent combined therapy.^28^

**Figure 6:**
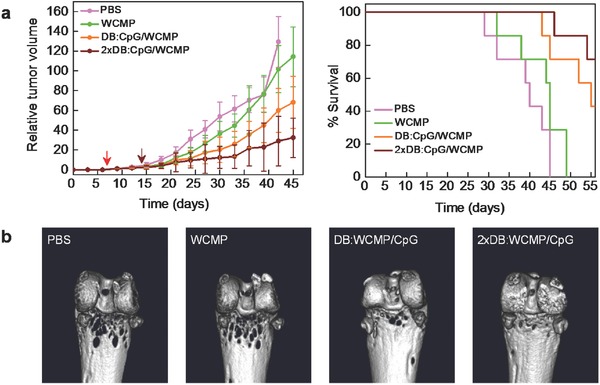
Therapeutic effect of biomimetic vaccine on breast cancer. a) Tumor growth and the survival time of the 4T1 breast tumor‐bearing mice after different treatments. Mice with established tumors were treated with PBS, WCMP, DB:CpG/WCMP, or 2 × DB:CpG/WCMP. Tumor progress and the survival time were recorded. Day 0 was the day of tumor transplantation, and arrows indicate days of prime (red) and booster (wine) vaccination, respectively. The data represent the mean ± SD with *n* = 7. b) Representative bone erosions caused by tumor metastasis after different treatment on day 35.

Encouraged by these remarkable antitumor performances, we next extended the DB‐based biomimetic system on the therapy of 4T1 breast cancer. Since tumor cell membrane presents a complex antigenic profile, the membrane‐bound tumor antigens have been used to train the immune system to recognize and fight cancers.^29^, ^30^ Thus, in this tumor model, instead of OVA, the whole cell membrane proteins (WCMP) extracted from 4T1 cells were used as the tumor antigen. As shown in **Figure** **6**a, immunization with DB:CpG/WCMP delayed the tumor growth. With one booster immunization, the tumor progress could be further inhibited with a significantly improved lifespan. Considering the highly metastatic property of 4T1 mammary adenocarcinoma, we also monitored the bone metastasis after different treatments (Figure 6b). Compared with the varied homolateral tibia erosions in other groups, the 2 × DB:CpG/WCMP group remain compact and smooth, again demonstrating satisfactory metastatic prevention. The analysis of antinuclear antibodies and anti‐ssDNA antibodies in Figure S8 (Supporting Information) also confirmed the safe use of our DB‐based biomimetic vaccine system.

### 2.7 The Biomimetic Vaccine Elicits Long‐Term Immune Memory and Effective Tumor Prevention

**Figure 7:**
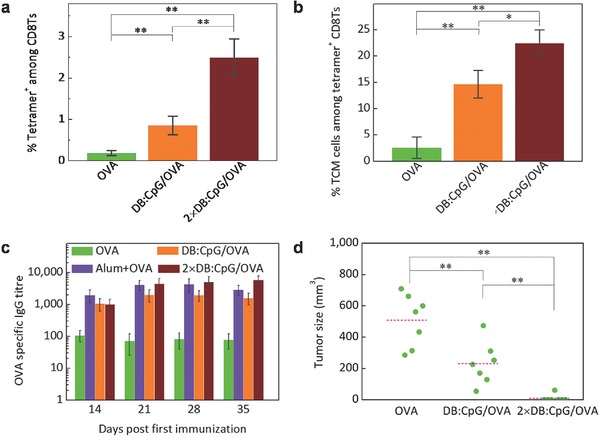
Biomimetic vaccine elicits effective immune memory and tumor prevention. C57BL/6 mice were subcutaneously immunized on day 0 with 20 µg of OVA or an equivalent dose of OVA formulated as DB:CpG/OVA, and the booster vaccination for the 2 × DB:CpG/OVA group was given on day 7. a) Frequency of OVA‐specific T cells (tetramer+) among CD8Ts in the LNs on day 28. b) Analysis of TCM cells among OVA‐specific CD8Ts by CD44/CD62L staining. c) ELISA of the total OVA‐specific IgG in the serum. A group that was immunized with Alum + OVA was used as a control. d) Mice immunized with OVA, DB:CpG/OVA, or 2 × DB:CpG/OVA were challenged with 1 × 10^6^ E.G7 cells on day 21. Tumor progression was monitored thereafter, and the presented data are tumor volumes on day 35. Data in parts (a)–(c) represent the mean ± SD (a, b: *n* = 3, c: *n* = 7). *p < 0.05, **p < 0.01.

Having achieved satisfactory therapeutic effects, we evaluated the protective effect of the biomimetic vaccine. Analysis of CD8Ts in LNs showed that the biomimetic vaccine potently promoted an endogenous OVA‐specific CD8T response, leading to a greatly increased proportion of OVA peptide–MHC tetramer + CD8Ts (**Figure** **7**a). More importantly, immunization with DB:CpG/OVA increased the frequency of central memory T (TCM) cells to 22.5% by 21 d after the booster vaccination, in contrast to 2.6% in the OVA group (Figure 7b). As TCM cells mediate long‐term cellular immunity, this result reflected the systemic activation of a persistent adaptive immune response.^31^ Moreover, the biomimetic vaccine elicited a strong and durable OVA‐specific IgG response. As shown in Figure 7c, immunization with OVA induced a barely detectable antibody response against OVA. By contrast, immunization with DB:CpG/OVA elicited durable anti‐OVA serum IgG levels, with a high titer, by day 35. A booster injection led to an even stronger IgG response, comparable with a response to the adjuvant aluminum hydroxide (alum).^32^, ^33^ The formation of strong immune memory in the form of both memory T cells and IgG indicated a hopeful outlook for tumor prevention. As expected, after challenge with E.G7 cells, six of seven mice in the 2 × DB:CpG/OVA group remained tumor free, whereas a heavy tumor burden was observed in all mice in the OVA group (Figure 7d), demonstrating the prominent prophylactic effect of the biomimetic vaccine against malignant tumors.

## 3 Conclusion

In summary, we developed a facile strategy to prepare DB and to construct a biomimetic vaccine with multiple pathogen‐like features. Taking advantage of intrinsic PAMPs, encapsulated CpG, and bacterial morphology, the biomimetic vaccine could potently recruit APCs, deliver/cross‐present antigens, and activate T cells, thus triggering robust cellular and humoral responses. Animal experiments demonstrated that effective tumor therapy, antimetastatic effects, and excellent tumor prophylaxis could be achieved. In the future, we will prepare DB from varying microorganisms using the current strategy and will screen for other candidates with high performance. Considering the facile guest component loading, we will also test the universality of the biomimetic vaccine formulation by applying defined tumor‐associated antigens and other pathogen‐derived immunomodulatory agents, promoting a further step toward clinical application.

## 4 Experimental Section

*Shape Selection, DB Fabrication, and Vaccine Preparation*: Four kinds of lactic acid bacteria (*P. acidilactici*, *S. thermophilus*, *L. casei*, and *B. adolescentis*) were first pretreated with 1% (v/v) formaldehyde overnight, and then washed three times with ultrapure water before hydrothermally treated at 150 °C within 6 h in ultrapure water. DB with ideal porosity were prepared with a combined pretreatment of 1% (v/v) formaldehyde and 1% (v/v) Triton X‐100, and then treated with a 6 h hydrothermal process at 150 °C after three times wash with water. To estimate their MR‐binding ability, the DB were consecutively incubated with recombinant murine CD206 (R&D) and an Alexa Fluor 488‐conjugated antimouse CD206 mAb (BioLegend). The binding amounts were determined by flow cytometry (FC; Beckman) analysis. To avoid nonspecific binding, 1% (v/v) bovine serum albumin was added prior to and during the incubation process. For biomimetic vaccine construction, CpG 1826 (5′‐TCCATGACGTTCCTGACGTT‐3′; TAKARA) and OVA (Sigma‐Aldrich) were loaded successively. Repeated washing with water was carried out to remove unloaded CpG and OVA. For CLSM imaging and FC analysis, HiLyte Fluor 647 amine (AnaSpec)‐labeled DB, AMCA‐labeled CpG (TAKARA), and fluorescein isothiocyanate (FITC; Amresco)‐labeled OVA were used instead of pure CpG and OVA.

*Interaction with DCs*: To study the biocompatibility of the hydrothermal treated bacteria, the cck8 test was carried out to evaluate the cytotoxicity of DB. To study the effect of bacteria's shape on DC internalization, the intracellular amounts were detected by flow cytometry (LSRFortessa, BD) utilizing their internal fluorescence within 405 nm excitation. Equal amount of hydrothermal treated bacteria with different shapes was incubated with DCs for 24 h at a series of concentrations, respectively. To study the affinity between the DB and DCs, binding forces were determined using a quantitative optical tweezers platform (NanoTracker, JPK). Briefly, individual DB were trapped by the tweezers and brought into contact with a DC. About 5 s later, the individual bacterium was moved away from the DC surface at a constant speed of 0.5 µm s^−1^, and the force of the interaction was recorded during this process. To study DC uptake of the DB, the intracellular amounts of DB were analyzed by FC after incubation with Hilyte‐647 dye‐labeled 0h‐9hDB (2.5 µg mL^−1^) for 24 h. To distinguish MR‐mediated uptake, DCs were incubated in advance with 200 µg mL^−1^ mannose for 1 h to block the MRs. For CLSM imaging, the cell membrane and cell nucleus were stained with rhodamine phalloidin (Invitrogen) and Hoechst 33 258 (Molecular Probes), respectively. To study the intracellular trafficking of the DB, early endosomes were immunofluorescently stained with polyclonal rabbit antimurine early endosome antigen‐1 (EEA1; Abcam) and Texas Red anti‐rabbit IgG (KPL), and lysosomes were stained with LysoTracker Red (Invitrogen). The corresponding fluorescent images were acquired with a spinning‐disk confocal system (UltraVIEW VoX, PerkinElmer).

*In Vivo T Cell and Antibody Activation*: The in vivo proliferation of OVA‐specific CD8Ts was measured by a dye dilution assay. Briefly, OT‐1 CD8Ts were stained with 0.5 × 10^−6^ m carboxyfluorescein diacetate succinimidyl ester (CFSE; Invitrogen), and 2 × 106 labeled OT‐I T cells were intravenously injected into C57BL/6 recipients. These mice were immunized with different formulations 12 h later. After an additional 72 h, the LN cells and splenocytes were extracted and stained with PerCP‐Cy5.5‐CD3 (eBioscience) and PE‐Cy7‐CD8α mAbs (BioLegend). The division of OVA‐specific CD8Ts was assessed by FC analysis of CFSE dilutions.

To evaluate the cytotoxic activity and memory effect of CD8Ts, single‐cell suspensions from the pooled spleens (*n* = 3) and LNs (*n* = 3) of immunized mice were isolated 28 d after the first vaccine injection. To assess antigen‐specific CTL activity, the splenocytes were restimulated using the peptide SIINFEKL (GL Biochem) for 3 d in PRIM 1640 media (Life Technologies) containing 20 U mL^−1^ recombinant IL‐2 (Cell Signaling Technology). Subsequently, these activated effector cells were mixed with mitomycin‐treated E.G7 cells (OVA‐transfected EL‐4 cells; ATCC) or EL‐4 target cells (ATCC). The CTL activity was evaluated at various ratios of effector cells to target cells (E/T ratios) in an LDH cytotoxicity detection assay (TAKARA). To evaluate effector memory and central memory phenotypes in LN cells, these cells were analyzed by FC after staining with SIINFEKL/H‐2Kb peptide–MHC tetramers (Becton Dickinson) and PerCP‐Cy5.5‐CD3, PE‐Cy7‐CD8α, APC‐Cy7‐CD44, and Pacific‐Blue‐CD62L mAbs (BioLegend). To evaluate the serum antibody level, blood samples were collected on days 14, 21, 28, and 35, and anti‐OVA IgG levels were determined by enzyme‐linked immunosorbent assay (ELISA) analysis. A vaccine formulated with 500 µg of alum adjuvant (aluminum hydroxide gel; Hualan Biological Engineering Incorporation) and 20 µg of soluble OVA was used as a positive control. To assess the proportion of IFN‐γ + CD8Ts, the splenocytes were stimulated ex vivo with 5 µg mL^−1^ SIINFEKL for 6 h with GolgiPlug (BD Bioscience). The cells were then fixed, permeabilized, stained with anti‐IFN‐γ and anti‐CD8α, and analyzed by FC.

*Quantities' Determination of CTL and Treg Cell in Tumor*: To determine the frequencies of CTL and Treg in tumor, the tumor tissues of mice in different groups were collected on day 21. Cell suspensions were then prepared by collagenase treatment and mechanical disruption. For flow cytometry analysis, FITC‐IFN‐γ mAb, and SIINFEKL/H‐2K^b^ peptide–MHC tetramers were used to label CTLs, while Pacific Blue‐CD4, PE‐CD25, and Alexa 488‐Foxp3 mAbs were used to label Treg cells. To observe the distribution of CTLs in tumor microenvironment, histological sections stained by FITC‐IFN‐γ mAb and Hoechst were imaged using CLSM.

*Vaccination Study*: To establish the EG.7‐tumor model, 1 × 10^6^ E.G7 cells were injected into the left axillary region of C57BL/6 mice on day 0. Vaccines were subcutaneously injected into the lower right flank. In each group, 20 µg of soluble OVA or an equivalent dose of OVA loaded with or without 2 µg of CpG into 50 µg of DB was used, and the same dose was adopted for the booster immunization. To assay the treatment of an established tumor, the immunization was given on day 7, when the tumor was palpable, and a booster immunization was administered to the 2 × DB:CpG/OVA group on day 14. For antimetastatic evaluation, tumor‐bearing animals that had undergone the same vaccination procedure were again challenged with 2 × 10^5^ E.G7 or EL‐4 cells in the right axillary region 7 d after the last vaccination. To assay early tumor therapy, mice were vaccinated 4 and 11 d (booster injection) after E.G7 cell transplantation. To assay tumor prevention, mice were vaccinated on days 0 and 7 (booster immunization) and challenged with 1 × 10^6^ E.G7 cells on day 21. To monitor tumor progression, tumor sizes were measured every another day and represented as 1/2 × *L* × *W* ^2^ (mm^3^). The tumor volumes of deceased mice were not included after the day of death.

To establish 4T1 mammary carcinoma, 1 × 10^6^ 4T1 cells were injected into the left second mammary fat pad of BALB/c mice on day 0. To prepare 4T1 vaccine, WCMP were extracted from 4T1 cells by using a Membrane and Cytosol Protein Extraction Kit (Beyotime). About 20 µg of soluble WCMP or an equivalent dose of WCMP loaded with 2 µg of CpG in 50 µg of DB was subcutaneously injected. The same treatment protocol as that of E.G7 tumor‐bearing mice was adopted. The bone erosions caused by tumor metastasis were imaged by using a microCT scanner (Quantum FX, Caliper Life Sciences). The imaging was carried out on a weekly basis for 4 weeks after the first treatment.

All animal experiments were performed in compliance with the guide of care and use of laboratory animals. Further experimental details are provided in the Supporting Information.

*Statistical Analysis*: Results were expressed as means ± standard deviation (SD), differences between two groups were tested using an unpaired, one‐tailed Student's *t*‐test. Differences among more than two groups were evaluated by one‐way analysis of variance (ANOVA) with significance determined by Tukey‐adjusted *t*‐tests.

## Conflict of Interest

The authors declare no conflict of interest.

## Supporting information

Supplementary
