# Supplementary material for: Biomimetically Engineered Demi‐Bacteria Potentiate Vaccination against Cancer
Source: Adv Sci (Weinh). 2017 Jun 15;4(10):1700083. doi: 10.1002/advs.201700083 (PMC5644226; doi:10.1002/advs.201700083)
Supplement: Supplementary file 1 — Supplementary [file ADVS-4-na-s001.pdf]

Copyright WILEY-VCH Verlag GmbH & Co. KGaA, 69469 Weinheim, Germany, 2017.

ADVANCED SCIENCE
Open Access

Supporting Information

for *Adv. Sci.,* DOI: 10.1002/advs.201700083

Biomimetically Engineered Demi-Bacteria Potentiate Vaccination against Cancer

Dezhi Ni, Shuang Qing, Hui Ding, Hua Yue, Di Yu, Shuang Wang, Nana Luo, Zhiguo Su, Wei Wei, and Guanghui Ma**

Copyright WILEY-VCH Verlag GmbH & Co. KGaA, 69469 Weinheim, Germany, 2016.

Supporting Information

Biomimetically engineered demi-bacteria potentiate vaccination against cancer Title

Dezhi Ni, Shuang Qing, Hui Ding, Hua Yue, Di Yu, Shuang Wang, Nana Luo, Zhiguo Su, Wei Wei and Guanghui Ma**

Experimental Section

Characterization of Surface Properties.

Zeta potential of the 4 kinds of hydrothermal treated bacteria was determined by ZetaSizer (Nanoseries, Malvern) in pure water and the surface contact angle was measured by using contact angle meter (OCA-20, DataPhysics).

Evaluation of Loading Efficiency and Release behaviour for OVA and CpG.

For the encapsulation studies, CpG or OVA at certain concentrations was mixed with 50 μg DB in 100 μL PBS at 4 ^{o}C for 12 h after vacuum negative pressure treated. Free CpG or OVA was washed away by three centrifugation-redispersion cycles, with all supernatants reserved. The loading amount of CpG or OVA was determined by mass balance between the initial incubation solution and the reserved supernatant. For in vitro release studies, OVA or CpG-loaded DB was incubated in 1 mL PBS (pH 7.4) under agitation at 37 °C. Supernatants were periodically collected by centrifugation and replaced with fresh buffer of equal volume. The CpG concentration was determined using an Infinite M200 microplate spectrophotometer and a NanoQuant Plate (Tecan), and the OVA concentration was determined by a BCA protein determination kit (Thermo).

Culture of DCs.

DCs were generated by flushing tibia and femurs of 6-8 weeks old male C57BL/6 mice. After red blood cell lysis, cells were cultured in complete RPMI 1640 medium (Life technologies) containing 20 ng mL^{-1} granulocyte macrophage colony- stimulating factor (GM-CSF, Peprotech), 10 ng/mL intereukin-4 (IL-4, Peprotech), and 10% (v/v) fetal bovine serum (FBS) for 6-8 days. FC Analysis after APC-Cy7-CD11c mAb (Biolegend) staining verified that a purity above 90% could be achieved.

Observation of Capture of DB at the DC Membrane.

For SEM imaging, DCs were adhered to poly-L-lysine-coated glass slides, and 2.5 μg mL^{-1} of DB was then added for 1 h of incubation. The cells were washed thoroughly immediately afterward to remove free DB. Next, the samples were fixed in 2.5% (v/v) glutaraldehyde, dehydrated with gradient methyl cyanide solutions, and gold-sprayed for imaging under a field emission SEM (JEOL JSM6700F).

Intracellular Trafficking of DB.

DCs were incubated with DB for 1 h at 37 ^{o}C and washed to remove free DB, followed by further incubation for another 6 h to ensure the synchronisation of intracellular processes. For early endosome staining, the cells were fixed in 4% (v/v) formaldehyde and permeabilized *via* a 10 min incubation with 0.2% (v/v) Triton X-100 at room temperature. Subsequently, 5% (v/v) goat serum was added and incubated for 30 min to block nonspecific adsorption. Staining with a polyclonal rabbit anti-murine EEA1 antibody was performed at 4 °C for 24 h, followed by 30 min of incubation with Texas Red-goat anti-rabbit IgG at room temperature. For lysosome staining, DCs were directly stained with LysoTracker Red for 30 min at 37 ^{o}C, without cell fixation and permeabilization. The colocalization ratios (CRs) of DB with early endosomes or liposomes were calculated using Leica LAS Colocalization software.

Preparation and Characterization of Traditional Particulate Adjuvants.

Liposomes were prepared by thin-film hydration method.[1] Briefly, a mixture of 1-palmitoyl-2-oleoylphosphatidylcholine (POPC) and cholesterol (in molecular ratio 1:2) were dissolved in chloroform, and a thin-film was formed on the inner side of the round bottom flask by evaporating the solvent under vacuum using a rotavapor. The film was then hydrated using 10 mL of PBS containing 50 ng mL^{-1} water-soluble Cy5. The formed liposomes were then sonicated using a probe sonicator for 2 min, and then sized by extrusion through a 0.4 μm pore-sized polycarbonate membranes repeatedly. The un-encapsulated Cy5 was then removed by dialysis. PLA NPs and Chitosan NPs were prepared using the Shirasu Porous Glass (SPG)

membrane emulsification technique.[2] By selecting a SPG membrane with a certain pore size, the size of the emulsion droplets and subsequent NPs can be well controlled around 500 nm. To prepare the fluorescent NPs, oil-soluble Cy5 was added together with PLA to the oil phase before the emulsification, free Cy5 was then removed by utterly washed with water. The zeta potential and size distribution were determined by Zetasizer (Nanoseries, Malvrn).

Evaluation of DC Activation and Antigen Presentation.

In total, 1×10^{6} cell mL^{-1} DCs were incubated with different formulations for 24 h in 24-well plates. At the end of the incubation, the DCs were collected by centrifugation (500 g, 3 min) and stained with antibody for 30 min at 4 ^{o}C. The following fluorophore-conjugated antibody reagents (purchased from BioLegend and eBioscience) were used: APC-Cy7-CD11c, PE-CD40, FITC-CD80, PE-CD86, PerCP-eFluor 450-MHC II, and APC-SIINFEKL-MHC I. Subsequently, the cells were washed with PBS, and the levels of surface costimulatory molecules and recognition signals for T cell activation were analysed by FC. Otherwise, to monitor the cytokine profile of the DCs, cell culture supernatants were collected, and the secretion levels of IL-6, IL-12, TNF-α, MCP-1, and IFN-γ were detected using a CBA Mouse Inflammation Kit (BD) to quantify.[3] Briefly, capture beads and phycoerythrin (PE) detection reagent were incubated with standard samples or test samples for 2 hours, washed in wash buffer. After acquisition of sample data using the flow cytometer, the sample results were tabulated and graphed using the BD CBA Analysis Software.

Analysis of PRR mRNA Expression.

RNA was isolated with TotalRNAExtractor (Sangon Biotech, Shanghai, China) from naive DCs and DC treated by DB, CpG, or DB:CpG for 24 h. RNA samples were then DNase I digested, reverse transcribed with iScript cDNA Synthesis Kit (Bio-Rad Laboratories, Hercules, Canada), and analysed by qPCR with Ssofast EVI Green Supermix (Bio-Rad Laboratories, Hercules, Canada). Data was displayed as relative expression compared to

GAPDH. PCR products were sequence verified. The sequences of primers for target genes and internal control gene GAPDH are listed in the table below.

Table. The sequences of primers for target genes and internal control gene GAPDH

	**Forward Primer**	**Reverse Primer**
**TLR2**	CCAGACACTGGGGGTAACATC	CGGATCGACTTTAGACTTTGGG
**TLR4**	AAAGTGGCCCTACCAAGTCTC	TCAGGCTGTTTGTTCCCAAATC
**TLR9**	GCTTTGGCCTTTCACTCTTG	AACTGCGCTCTGTGCCTTAT
**NOD2**	CAGGTCTCCGAGAGGGTACTG	GCTACGGATGAGCCAAATGAAG
**MRC1**	GGTTATGAAAGGCAAGGATGGA	TTGTCTGCACCCTCCGGTACTA
**MyD88**	CACCTGTGTCTGGTCCATTG	AGGCTGAGTGCAAACTTGGT
**GAPDH**	CTCATGACCACAGTCCATGC	CACATTGGGGGTAGGAACAC

***In-vivo* Fate of Antigen.**

To observe antigen distribution *in vivo*, OVA was labelled by near-infrared fluorescence probe Cy5 (Fanbo Biochemicals). 20 μg free or DB-loaded Cy5-OVA were s.c. administrated at the hindneck of C57BL/6 mice. The *in vivo* distribution of fluorescence labelled OVA and the relative fluorescence intensities were recorded at certain intervals by In Vivo Imaging System FX Pro (Kodak). For a histological analysis of cell recruitment at the vaccine site, mice were sacrificed after injected different vaccine formulations, at the indicated time points, the injection site was excised and fixed in 3.7% (v/v) formaldehyde. These tissues were processed for histological examination by hematoxylin and eosin (H&E) staining at the Peking University Health Science Center. The optical images of the tissue slices were taken by Vectra platform and the recruited cells were quantitated by using the inform software (Caliper Life Sciences, Hopkinton, USA).[4]

Analysis of DC Migration to Lymph Nodes.

To track *in vivo* DC migration from injection site towards lymph nodes, the draining

lymph nodes (LNs) were harvested at 24 h after immunisation. Cell suspensions from LNs were prepared by mechanical disruption and pressing of the tissue through 70 μm cell strainers. The CD11c$^+$ SIINFEKL-MHC I$^+$ cell numbers among CD11c$^+$ cells (DCs) were examined after staining by APC-Cy7-CD11c and APC- SIINFEKL-MHC I mAbs.

Preparation of Splenocytes and CD8 T Cells.

Spleen of 6-8 weeks old male C57BL/6 or OT-1 mice was harvested and ground to prepare single-cell suspension. After red blood cell lysis, cells were washed and cultured in complete RPMI 1640 medium containing 10% (v/v) heat-inactivated FBS. For CD8 T cell enriching, splenocytes were sorted by Dynabeads (Life technologies) to deplete CD8-negative leucocytes, and CD8T with purity around 90% could be harvested.

Evaluation of Health Condition.

To examine the T cell-subset distribution after the treatment of developed tumors, mice that had received different treatments were sacrificed on day 23, and LN cells and splenocytes were extracted; stained with PerCP-Cy5.5-CD3, PE-Cy7-CD8α, and Alexa Fluor 700-CD4 mAbs (all purchased from eBioscience); and analysed using FC. To determine the serum biochemical parameters, blood samples were collected before sacrifice for tumor-bearing mice or 35 days after the first immunisation for normal mice. The AST, ALT, BUN, ALP, and LDH levels were determined spectrophotometrically using an automated analyser (Hitachi-917, Hitachi Ltd., Tokyo, Japan). For a histological analysis of the effect on organs, the heart, liver, spleen, lung, and kidney were collected and fixed in 3.7% (v/v) formaldehyde. These tissues were processed for histological examination by hematoxylin and eosin (H&E) staining at the Peking University Health Science Center. The optical images of the tissue slices were taken by Vectra platform (Caliper Life Sciences, Hopkinton, USA). For body temperature observation, mice anal temperature was recorded using microprobe thermometer (Physitemp, BAT-12). For serum cytokine evaluation, sera were collected at different time points post administration. The serum levels of IL-6 were quantified using the ELISA kits according to

the manufacture's protocol (eBioscience).

Quantitative Determination of CTL and Treg Cell in Tumor.

To determine the frequencies of CTL and Treg in tumor, the tumor tissues of mice in different groups were collected on day 21. Cell suspensions were then prepared by collagenase treatment and mechanical disruption. For flow cytometry analysis, FITC-IFN-γ mAb and SIINFEKL/H-2K^b peptide-MHC tetramers were used to label CTLs, while Pacific Blue-CD4, PE-CD25, and Alexa 488-Foxp3 mAbs were used to label Treg cells. To observe the distribution of CTLs in tumor microenvironment, histological sections stained by FITC-IFN-γ mAb and Hoechst were imaged using CLSM.

Autoantibodies detection.

Serum harvested on day 14 and 28 after immunization with 4T1 vaccine, PBS was used as a control. Anti-nuclear antibodies (ANA) and anti-single-stranded DNA antibodies in serum were determined by ELISA according to the manufacture's protocols (Alpha Diagnostic International).

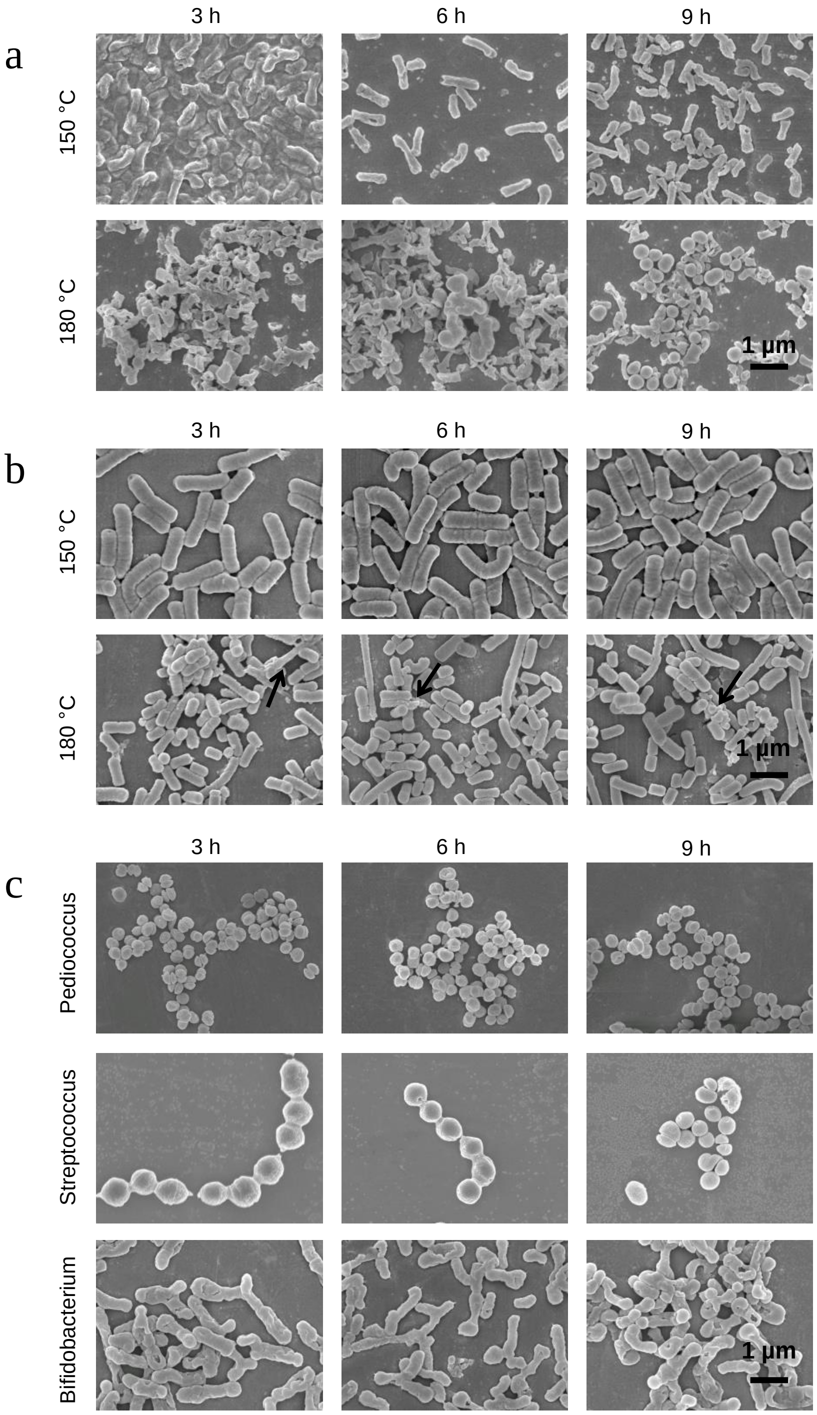

Figure S1. Effect of hydrothermal treatment on the bacterial morphology.
(a) Effect of hydrothermal conditions on the bacterial morphology, taking bacillus as an example. Disrupted structures were obtained following an increase in the hydrothermal treatment time or temperature, indicating that the cell wall of bacteria could not endure harsh hydrothermal treatment.
(b) Effect of cross-linking pretreatment on the bacillus morphology. In this case, 150 °C was regarded as the optimal temperature, considering a few fragments in the samples prepared 180 °C (as arrows indicated).
(c) Optimization of hydrothermal treatment for bacterial with other shapes. In this case, hydrothermal time was controlled within 6 h for the next comparative study as the chain structure of *Streptococcus* was fractured after 9 h treatment.

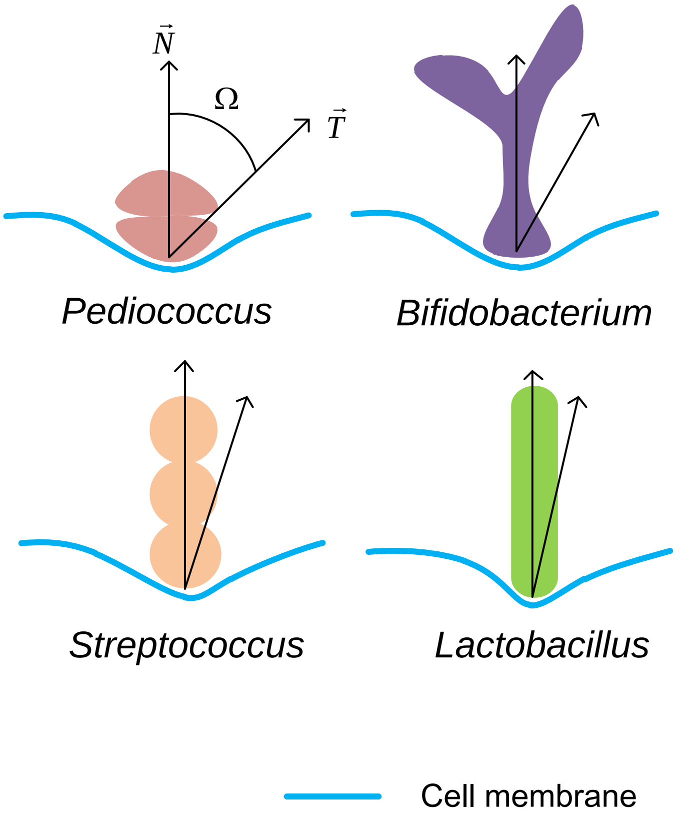

Figure S2. Definition of Ω.

$\vec{T}$ represents the average of tangential angles from 0° to 90°. Ω is the angle between $\vec{T}$ and cell membrane normal at the site of attachment, $\vec{N}$. As shown, the Ω of *Pediococcus* (spherical) was greatest while *Lactobacillus* (rod shaped) has the minimum angle among the 4 sets of bacteria.

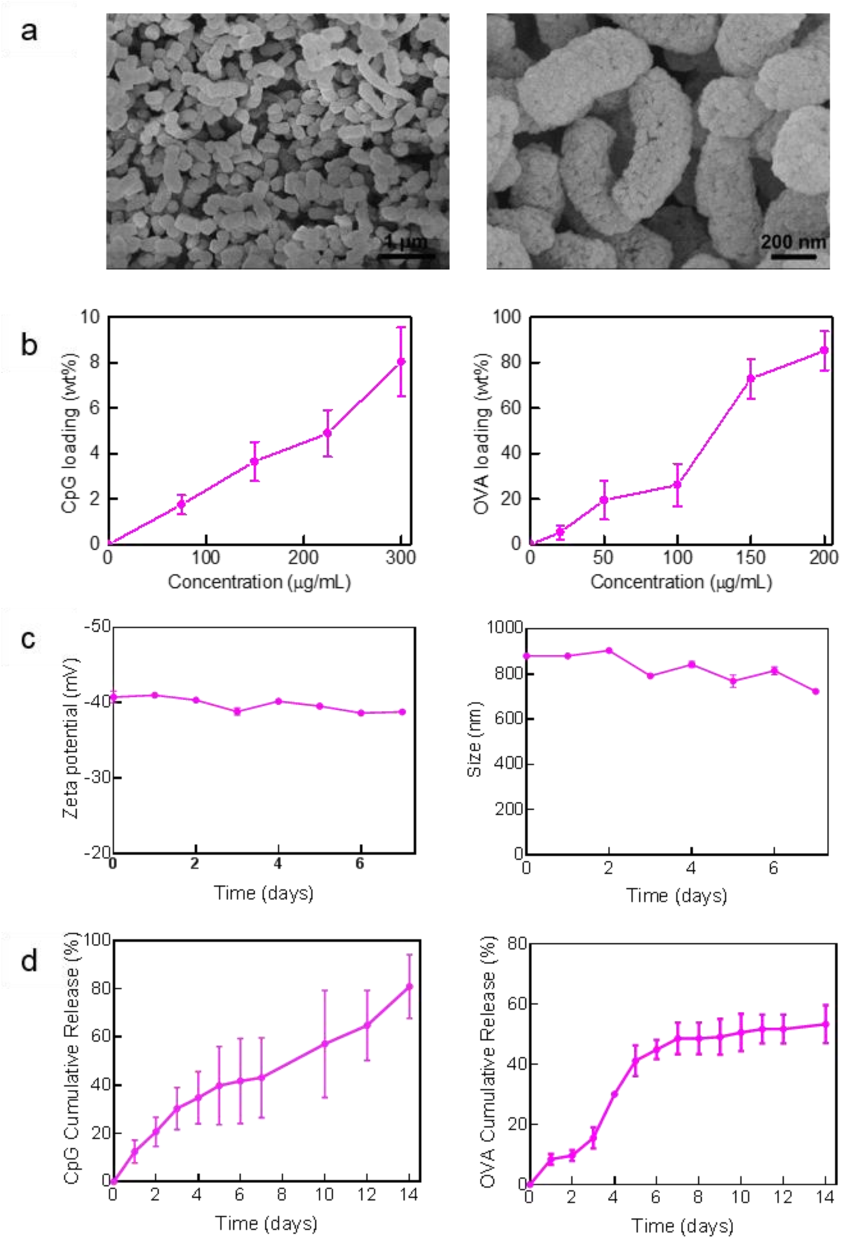

Figure S3. Preparation and characterization of DB with hollow/porous structure.
(a) Combined pretreatment with formaldehyde and Triton X-100 to obtain DB with hollow/porous structure.
(b) Loading efficiency of CpG and OVA within DB at different initial concentration. DB with ideal hollow interior/porous shell structure exhibits excellent guest-encapsulating ability, which enhanced subsequent antigen delivery with high efficiency.
(c) Stability investigation. Both the zeta potential and size remained stable during the investigation period, demonstrating good stability of our DB platform.
(d) Release profiles of loaded molecules *in vitro*. OVA and CpG sustainably released from the hollow/porous DB, which could protect antigen and CpG from rapid metabolism and diffusion in *vivo*.
The data represent the mean ± s.d. of three independent experiments with *n*=3

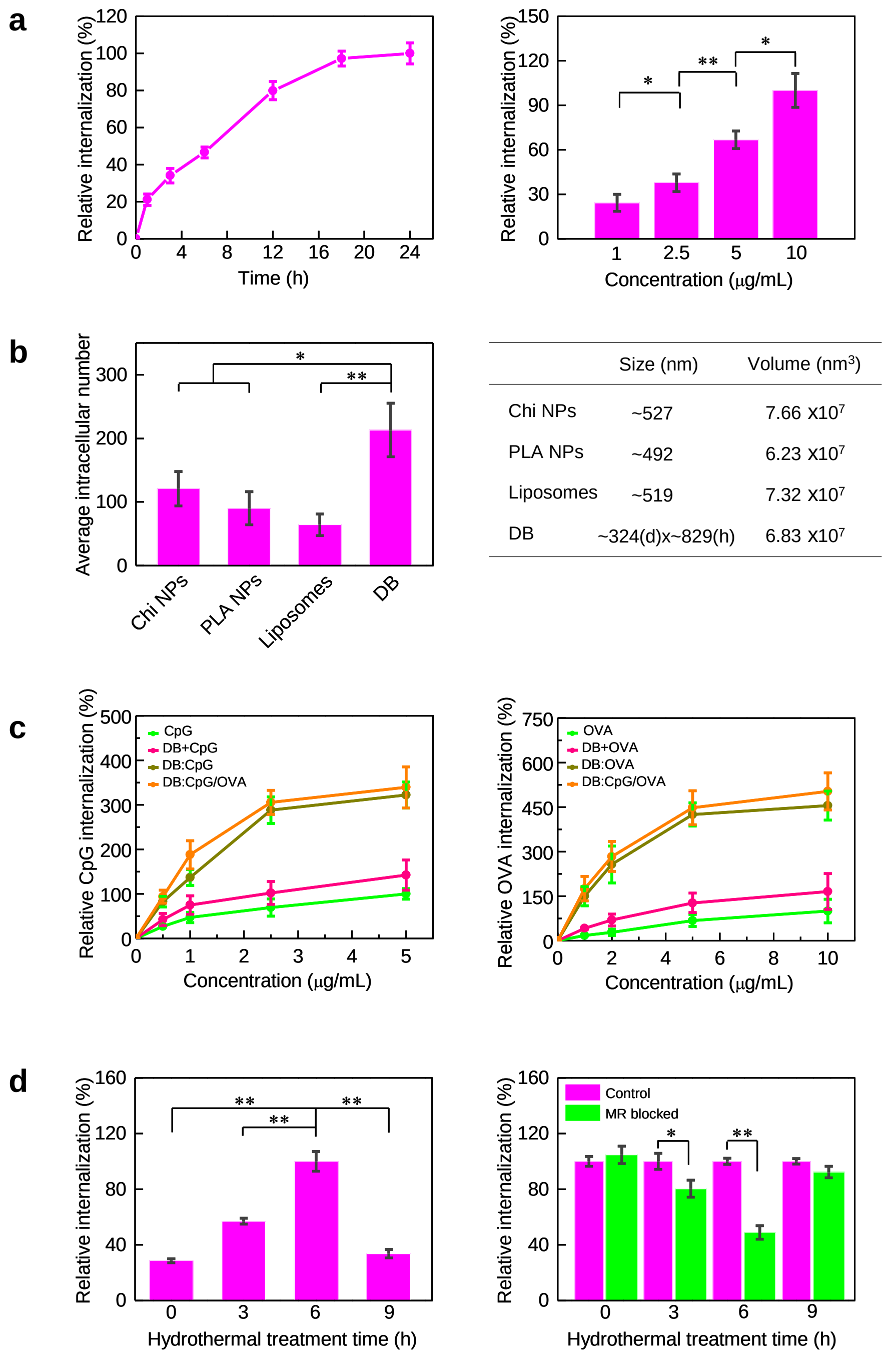

	Size (nm)	Volume (nm³)
Chi NPs	~527	7.66 X10^7
PLA NPs	~492	6.23 X10^7
Liposomes	~519	7.32 X10^7
DB	~324(d)x~829(h)	6.83 X10^7

Figure S4. Investigation of the internalization of DB with DCs.
(a) Time- and concentration-dependent internalization profiles of DB in DCs. The internalization of DB constantly increased as the initial concentration increased, indicating a favorable uptake of DB by APCs.
(b) Comparison of DC uptake amount between DB and traditional particulate adjuvants. In comparison with traditional adjuvant particles, chitosan nanoparticles (Chi NPs), poly (lactic acid) nanoparticles (PLA NPs), and liposomes with similar volumes to that of DB, DB exhibited the most potent DC uptake. The number of intracellular particle was calculated from equation - N (number of uptake) = T (total intracellular signal)/S (signal of particle individual). For all groups, the initial particle number in culture medium was normalized to that of DB (2.5 μg mL^{-1}).
(c) Comparative internalization profiles of free, DB-mixed, and DB-loaded CpG and OVA. DC uptake of both guest agents was found to be greatly improved by encapsulation in DB, enhanced intracellular delivery for both CpG and OVA could be achieved simultaneously.
(d) Effect of the MRs on the uptake of DB by DCs. Relative DC uptake of DB prepared *via* different hydrothermal treatment times was shown in left, to determine the proportion of MR-mediated endocytosis, mannose (200 μg mL^{-1}) was added to block the MRs 1 h before co-incubation. The data of MR-blocked group in were normalized to the internalization in the corresponding control group (left). These results confirmed that the MR ligand on 6hDB was sufficient to significantly promote the interaction with APCs.
Data in all groups represent the mean ± s.d. of three independent experiments with n=3. *$p<0.05$, **$p<0.01$.

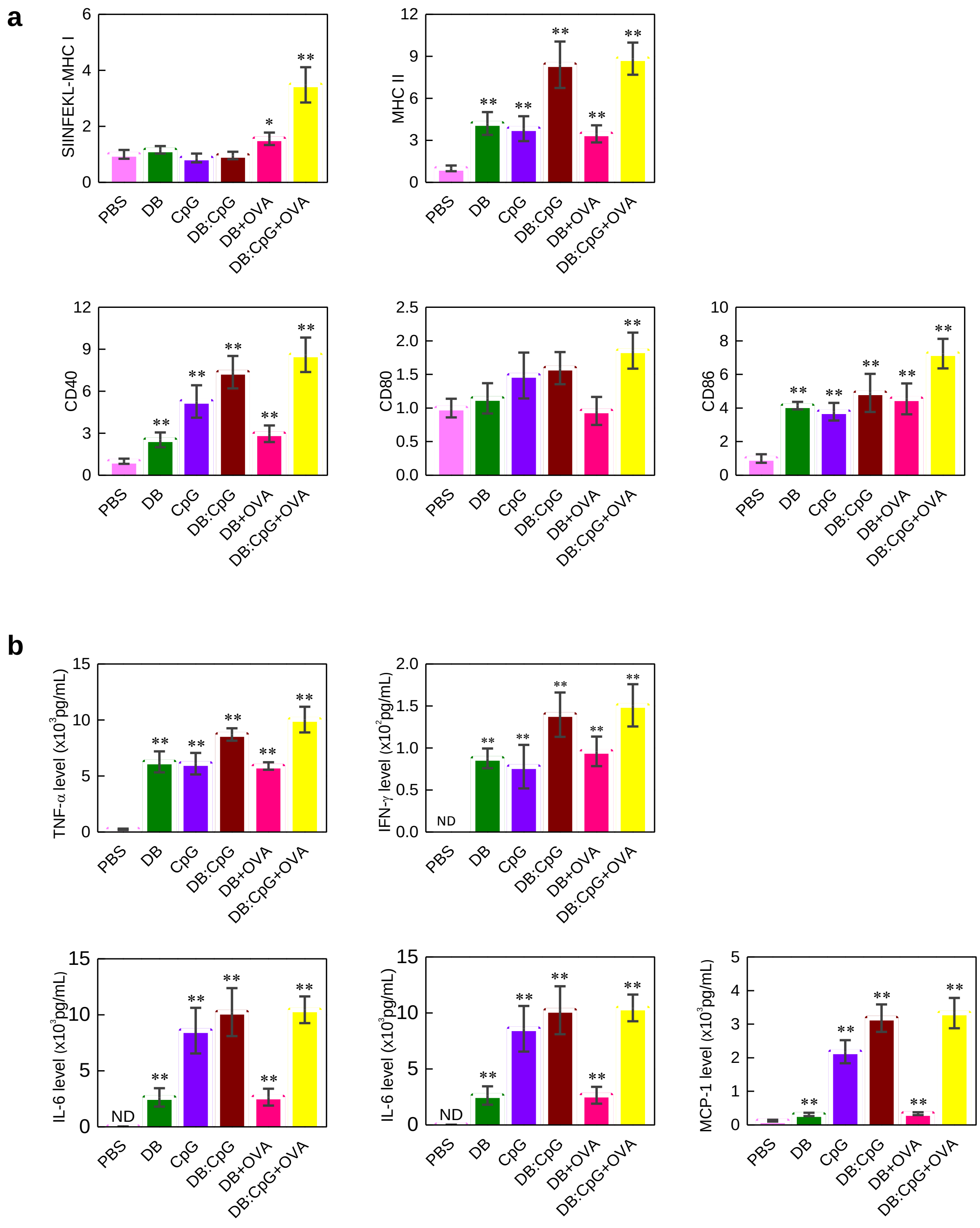

Figure S5. ***In vitro* DC stimulation.**
(a) Expression of recognition signals and costimulatory markers on DC surface after incubation with different formulations.
(b) Cumulative cytokine secretion of DC after incubation with different formulations. For all groups, 1 μg mL^{-1} of OVA, 100 ng mL^{-1} of CpG, and 2.5 μg mL^{-1} of DB were adopted. The data were normalized to the expression level of the PBS group.
Data represent the mean ± s.d. of three independent experiments with n=3. *$p<0.05$, **$p<0.01$.

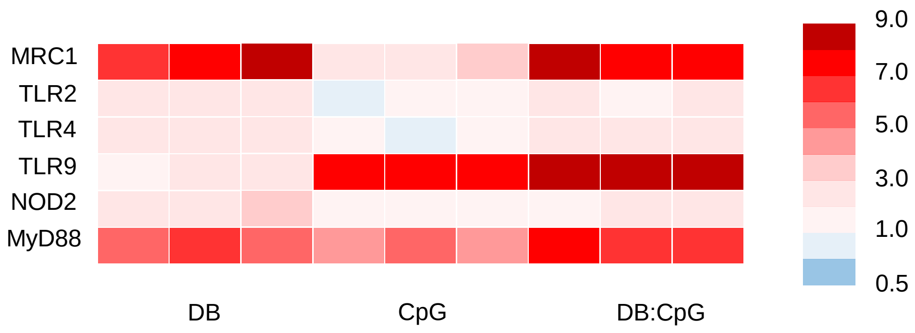

Figure S6. Heat-map representation of PRR mRNA expression in DCs after different treatments. The colour of the respective box in one row represents the expression value of the mRNA expression in one sample compared with the normal expression level in untreated DCs. Red, expression levels higher than normal; blue, expression levels below normal. The most distinct upregulation was found in the expression of mannose receptor mRNA MRC1 after exposure to DB, which further verified the predominant role of MR played during the DB-APC interaction. The efficient intracellular delivery of CpG molecules by DB encapsulation was also found to result in more drastic upregulation of TLR9 mRNA expression than sole CpG.

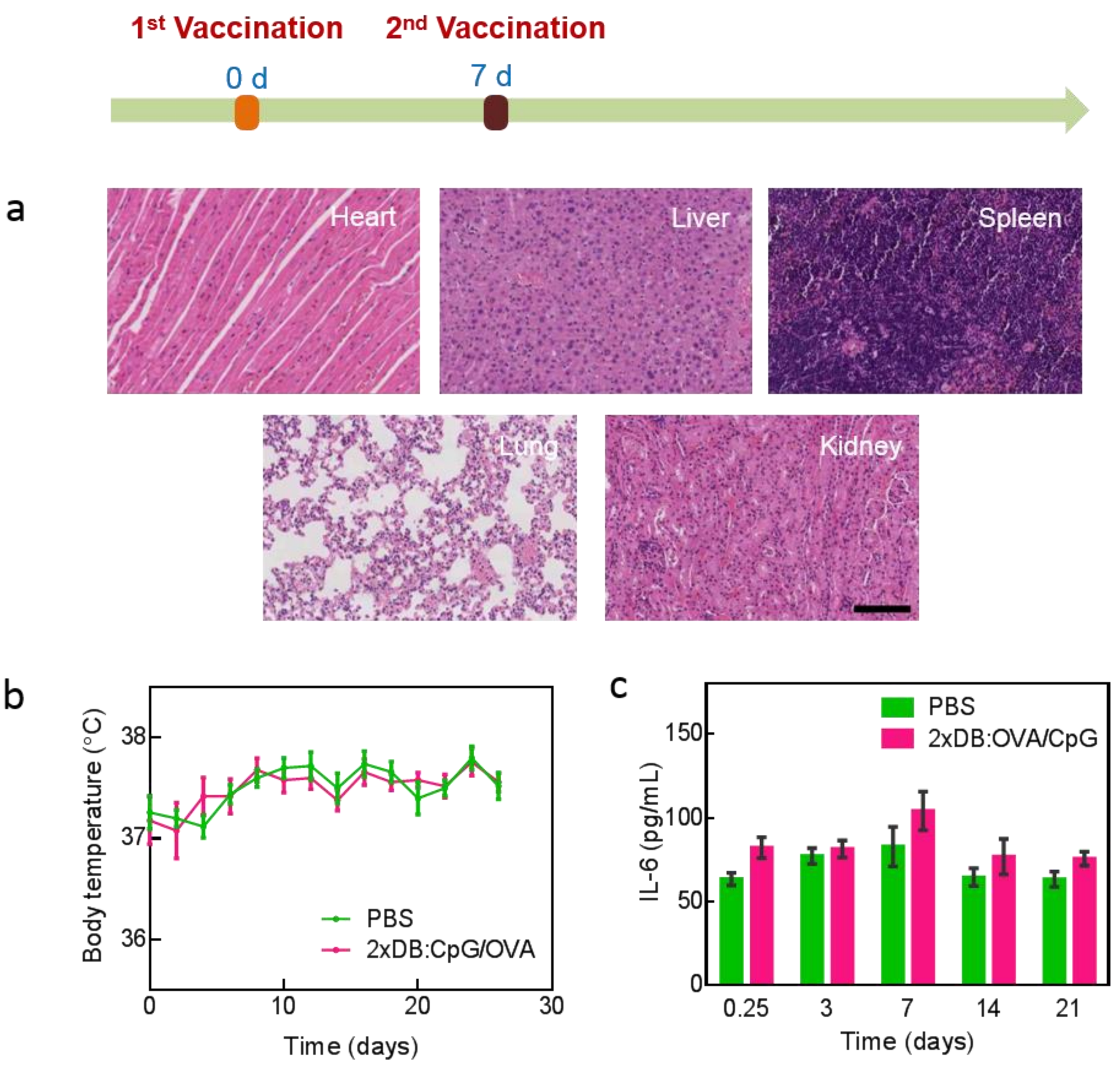

Figure S7. Biosafety investigation.
(a) Histological analysis. To evaluate organ toxicity, the main organs of the immunised mice were harvested 7 days after the second injection of DB:CpG/OVA. No organic damage or acute inflammation was observed in the histological sections of the heart, liver, spleen, lung, and kidney, confirming the safety of the biomimetic vaccine. Scale bar, 100 μm.
(b) Body temperature surveillance. No abnormal temperature was found in the treated group compared with that of the control group.
(c) Cytokine profile in serum. Serum levels of IL-6 at different time points after administration were quantified. Little difference was found between the treated group and PBS group, which further confirmed the good safety of our biomimetic vaccine. Data represent the mean ± s.d. with n=5.

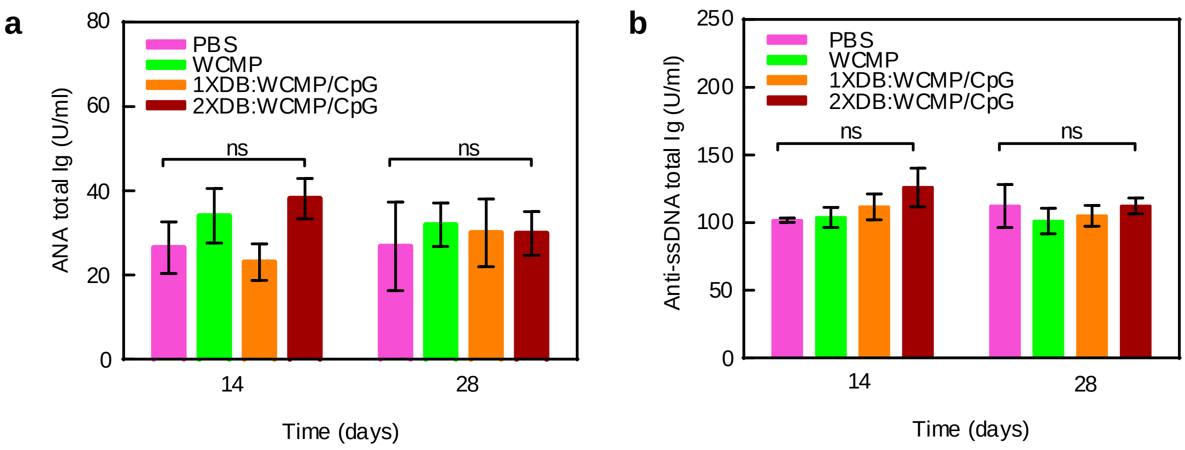

Figure S8. Analysis of autoantibodies in mice after injecting 4T1 WCMP based vaccine.
(a) The concentration of ANA total Ig detected in serum.
(b) The concentration of anti-ssDNA total Ig detected in serum.
ANA and anti-ssDNA antibodies are classic autoantibodies induced by cell proteins and single-stranded DNA, respectively. As shown, no statistically significant difference was observed either in ANA or anti-ssDNA antibodies compared with controlled group The data represent the mean ± s.d. with n=7. ns, $p>0.05$, not significant.

Table S1. Surface properties of different bacteria after hydrothermal treatment.

**Bacteria**	**Zeta potential**	**Contact angle (glycerinum)**	
Pediococcus	-24.03 mV	39.4°	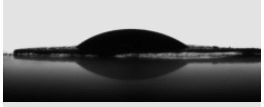
Bifidobacterium	-21.80 mV	39.3°	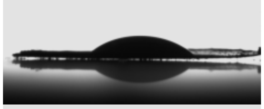
Streptococcus	-21.63 mV	41.7°	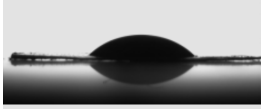
Lactobacillus	-22.63 mV	37.8°	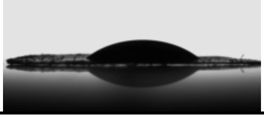

Table S2. Biocompatibility evaluation *via* serum biochemical parameters.
Healthy C57BL/6 mice were s.c. administered DB:CpG/OVA once or twice, and the toxicity effect was evaluated according to serum biochemical parameters, including aspartate aminotransferase (AST), alanine aminotransferase (ALT), blood urea nitrogen (BUN), lactate dehydrogenase (LDH), and alanine aminotransferase (ALP), 35 days after the first vaccination. The results showed that injection of the biomimetic vaccine caused no detectable abnormality in the above mentioned biochemical parameters, verifying the excellent biocompatibility of the biomimetic vaccine. The data are presented as the mean ± s.d. with n=7.

	**PBS**	**DB:CpG/OVA**	**2×DB:CpG/OVA**	**Normal range**
**AST**	104±63	92±41	93±37	54-298
**ALT**	40±24	45±22	50±26	17-77
**BUN**	12.66±5.34	12.21±4.38	12.16±4.07	8-33
**LDH**	799±175	826±190	821±148	215-1024
**ALP**	169±51	148±59	142±46	60-209

Table S3. Determination of the serum biochemical parameters of tumor-bearing mice. Mice with established tumors were treated with different formulations, blood samples were collected before scarification on day 23 (for developed tumor therapy), and the serum AST, ALT, BUN, LDH, and ALP levels were determined. The data are represented as the mean ± s.d. with n=7. Among these parameters, AST and ALT are specific indicators of hepatic toxicity, and LDH is a parameter related to injury of major organs, including the liver, heart, and kidney. In contrast with the abnormally enhanced levels in the other groups, the serum AST and ALT concentrations in the 2×DB:CpG/OVA group returned to normal ranges. A similar result was observed for the LDH levels, which indicated that treatment with the biomimetic vaccine had effectively protected the mice from hepatic or other organ damage. Together, all these data showed that mice that received the biomimetic vaccine were in much better health and had a better prognosis.

	**PBS**	**OVA**	**DB:OVA**	**CpG+OVA**	**DB:CpG/OVA**	**2×DB:CpG/OVA**	**Normal range**
**AST**	1366±310	1361±359	972±263	972±224	386±189	175±102	54-298
**ALT**	367±126	318±119	220±92	146±83	88±41	62±33	17-77
**BUN**	15.84±4.91	9.55±4.03	14.44±5.90	15.93±3.65	10.1±4.83	10.65±3.81	8-33
**LDH**	3514±752	3447±720	3072±713	3492±662	1744±403	753±168	215-1024
**ALP**	47±17	31±17	42±20	71±22	107±35	178±30	60-209

References:

[1] K. Muppidi, A.S. Pumerantz, J. Wang, G. Betageri. *ISRN Pharm.* **2012**, 2012, Article ID 636743.

[2] Z. Yue, Z. You, Q. Yang, P. Lv, H. Yue, B. Wang, D. Ni, Z. Su, W. Wei, G. Ma. *J. Mater. Chem. B* **2013**, 1, 3239.

[3] L. Trautmann, L. Janbazian, N. Chomont, E.A. Said, S. Gimmig, B. Bessette, M.-R. Boulassel, E. Delwart, H. Sepulveda, R.S. Balderas. *Nat. Med.* **2006**, 12, 1198.

[4] W. Huang, K. Hennrick, S. Drew. *Hum. Pathol.* **2013**, 44, 29.
